# Supplementary material for: Contribution of Reliable Chromatographic Data in QSAR for Modelling Bisphenol Transport across the Human Placenta Barrier
Source: Molecules. 2023 Jan 4;28(2):500. doi: 10.3390/molecules28020500 (PMC9863378; doi:10.3390/molecules28020500)
Supplement: Supplementary file 1 [file molecules-28-00500-s001.zip › molecules-2111370-supplementary-materials.pdf]

Supplementary Materials

Contribution of Reliable Chromatographic Data in QSAR for Modelling Bisphenol Transport across the Human Placenta Barrier

Clémence A. Gély [1,2], Nicole Picard-Hagen [1], Malika Chassan [2], Jean-Christophe Garrigues [3], Véronique Gayrard [1] and Marlène Z. Lacroix [2,*]

[1] ToxAlim (Research Centre in Food Toxicology), National Research Institute for Agriculture, Food and Environment (INRAE), National Veterinay School of Toulouse (ENVT), University of Toulouse, 31076 Toulouse, France
[2] Therapeutic Innovations and Resistances (INTHERES), National Research Institute for Agriculture, Food and Environment (INRAE), National Veterinay School of Toulouse (ENVT), University of Toulouse, 31076 Toulouse, France
[3] Molecular Interactions and Chemical and Photochemical Reactivity Laboratory (IMRCP), University of Toulouse, 31062 Toulouse, France
* Correspondence: marlene.lacroix@envt.fr

<u>R codes to carry out ANN, with input, the descriptors and output the clearance indices.</u>

```
library(neuralnet)
data=cbind.data.frame(input,output)
train=data$output
formule= paste(colnames(data[-ncol(data)]),collapse = "+")
print(formule)
formule=paste("train ~ ",formule,sep ="")
print(formule)
set.seed(100)
neuralnet=neuralnet::neuralnet(as.formula(formule),data=data,hidden = 0, linear.output = FALSE, stepmax = 100000, threshold = 0.005)
```

Table S1: Molecular descriptors used in QSAR development. Molecular descriptors for the 15 bisphenols are described in the Excel file supplementary data.

Molecular descriptors			
	**Molecular parameters**	**Units**	**Descriptor name**
**ChemPropPro**	**Molecular weight**	**g/mol**	**MM**
	**Boiling Point**	**Kelvin**	**CPP 1**
	**Critical Pressure**	**Bar**	**CPP 2**
	**Critical Temperature**	**Kelvin**	**CPP 3**
	**Critical Volume**	**cm^3/mol**	**CPP 4**
	**Gibbs Free Energy**	**kJ/mol**	**CPP 5**
	**Heat Of Formation**	**kJ/mol**	**CPP 6**
	**Henry's Law constant**		**CPP 7**
	**Ideal Gas Thermal Capacity**	**J/(mol.K)**	**CPP 8**
	**logP**		**CPP 9**
	**Melting Point**	**Kelvin**	**CPP 10**
	**Mol Refractivity**	**cm^3/mol**	**CPP 11**
**Log P**	**Log P Viswanadhan's fragmentation**		**LP 2**
	**Log P Broto's fragmentation**		**LP 3**
**MR**	**MR Crippen's fragmentation**	**cm^3/mol**	**MR 1**
	**MR Viswanadhan's fragmentation**	**cm^3/mol**	**MR 2**
**ChemPropStd**	**Connolly Accessible Area**	**Å^2**	**CPS 1**
	**Connolly Molecular Area**	**Å^2**	**CPS 2**
	**Connolly Solvent Excluded Volume**	**Å^3**	**CPS 3**
	**Number of HBond Acceptors**		**CPS 4**
**CLogP Driver**	**Mol Refractivity**		**CD 1**
	**Partition Coefficient**		**CD 2**
**Molecular Networks**	**Log P**		**MN 1**
	**Log S**		**MN 2**
	**pKa1**		**MN 3**
	**pKa2**		**MN 4**
**Molecular Topology**	**Balaban Index**		**MT 1**
	**Cluster Count**		**MT 2**
	**Molecular Topological Index**		**MT 3**
	**Num Rotatable Bonds**	**Bonds**	**MT 4**
	**Polar Surface Area**	**Å^2**	**MT 5**
	**Radius**	**Atoms**	**MT 6**
	**Shape Attribute**		**MT 7**
	**Shape Coefficient**		**MT 8**
	**Sum Of Degrees**		**MT 9**

	Sum Of Valence Degrees		MT 10
	Topological Diameter	Bonds	MT 11
	Total Connectivity		MT 12
	Total Valence Connectivity		MT 13
	Wiener Index		MT 14
Electronic descriptors	Heat of formation	kJ/mol	DE 1
	Total Energy	eV	DE 2
	Electronic Energy	eV	DE 3
	Core-core repulsion	eV	DE 4
	Cosmo Area	Å^2	DE 5
	Cosmo Volume	Å^3	DE 6
	Ionisation Potential	eV	DE 7
	Homo Energy	eV	DE 8
	Lumo Energy	eV	DE 9
	Dipole moment	debye	DE 10

Table S2: Chromatographic descriptors used in QSAR development. Chromatographic descriptors values for the 15 bisphenols are available in Excel file supplementary data.

Chromatographic descriptors			
Column / solvent	Chromatographic parameters	Units	Descriptor name
BEH C18 100*2.1 1.7 µm AcN	Width 5%	min	C18A 2
	Retention Factor /BPA		C18A 5
	Asymmetry		C18A 7
	Peak Tailing		C18A 8
CSH Phenyl hexyl 100*2.1 1.7 µm AcN	Width 5%	min	PHA 2
	Retention Factor /BPA		PHA 5
	Asymmetry		PHA 7
	Peak Tailing		PHA 8
CSH Fluoro Phenyl 100*2.1 1.7 µm ACN	Width 5%	min	FPA 2
	Retention Factor /BPA		FPA 5
	Asymmetry		FPA 7
	Peak Tailing		FPA 8
BEH C8 100*2.1 1.7 µm ACN	Width 5%	min	C8A 2
	Retention Factor /BPA		C8A 5
	Asymmetry		C8A 7
	Peak Tailing		C8A 8
BEH Phenyl 100*2.1 1.7 µm ACN	Width 5%	min	PA 2
	Retention Factor /BPA		PA 5
	Asymmetry		PA 7

	Peak Tailing		PA 8
HSS T3 100*2.1 1.8 µm ACN	Width 5%	min	T3A 2
	Retention Factor /BPA		T3A 5
	Asymmetry		T3A 7
	Peak Tailing		T3A 8
raptor biphenyl 100*2.1 2.7 µm ACN	Width 5%	min	RBA 2
	Retention Factor /BPA		RBA 5
	Asymmetry		RBA 7
	Peak Tailing		RBA 8
HSS PFP 100*2.1 1.8 µm ACN	Width 5%	min	PFPA 2
	Retention Factor /BPA		PFPA 5
	Asymmetry		PFPA 7
	Peak Tailing		PFPA 8
BEH RP18 Shield 100*2.1 1.7 µm ACN	Width 5%	min	RPA 2
	Retention Factor /BPA		RPA 5
	Asymmetry		RPA 7
	Peak Tailing		RPA 8
HSS CYANO 100*2.1 1.8 µm ACN	Width 5%	min	CNA 2
	Retention Factor /BPA		CNA 5
	Asymmetry		CNA 7
	Peak Tailing		CNA 8
Force Biphenyl 100*2.1 1.8 µm ACN	Width 5%	min	FBA 2
	Retention Factor /BPA		FBA 5
	Asymmetry		FBA 7
	Peak Tailing		FBA 8
Raptor (Corshell) Biphenyl 100*2.1 1.8 µm ACN	Width 5%	min	RCBA 2
	Retention Factor /BPA		RCBA 5
	Asymmetry		RCBA 7
	Peak Tailing		RCBA 8
Cortecs C18 + (corshell)100*2.1 1.6 µm ACN	Width 5%	min	CC18A 2
	Retention Factor /BPA		CC18A 5
	Asymmetry		CC18A 7
	Peak Tailing		CC18A 8
BEH C18 100*2.1 1.7 µm MeOH	Width 5%	min	C18M 2
	Retention Factor /BPA		C18M 5
	Asymmetry		C18M 7
	Peak Tailing		C18M 8
CSH Phenyl hexyl 100*2.1 1.7 µm MeOH	Width 5%	min	PHM 2
	Retention Factor /BPA		PHM 5
	Asymmetry		PHM 7
	Peak Tailing		PHM 8

CSH Fluoro Phenyl 100*2.1 1.7 µm MeOH	Width 5%	min	FPM 2
	Retention Factor /BPA		FPM 5
	Asymmetry		FPM 7
	Peak Tailing		FPM 8
BEH C8 100*2.1 1.7 µm MeOH	Width 5%	min	C8M 2
	Retention Factor /BPA		C8M 5
	Asymmetry		C8M 7
	Peak Tailing		C8M 8
BEH Phenyl 100*2.1 1.7 µm MeOH	Width 5%	min	PM 2
	Retention Factor /BPA		PM 5
	Asymmetry		PM 7
	Peak Tailing		PM 8
HSS T3 100*2.1 1.8 µm MeOH	Width 5%	min	T3M 2
	Retention Factor /BPA		T3M 5
	Asymmetry		T3M 7
	Peak Tailing		T3M 8
raptor biphenyl 100*2.1 2.7 µm MeOH	Width 5%	min	RBM 2
	Retention Factor /BPA		RBM 5
	Asymmetry		RBM 7
	Peak Tailing		RBM 8
HSS PFP 100*2.1 1.8 µm MeOH	Width 5%	min	PFPM 2
	Retention Factor /BPA		PFPM 5
	Asymmetry		PFPM 7
	Peak Tailing		PFPM 8
BEH RP18 Shield 100*2.1 1.7 µm MeOH	Width 5%	min	RPM 2
	Retention Factor /BPA		RPM 5
	Asymmetry		RPM 7
	Peak Tailing		RPM 8
HSS CYANO 100*2.1 1.8 µm MeOH	Width 5%	min	CNM 2
	Retention Factor /BPA		CNM 5
	Asymmetry		CNM 7
	Peak Tailing		CNM 8
Force Biphenyl 100*2.1 1.8 µm MeOH	Width 5%	min	FBM 2
	Retention Factor /BPA		FBM 5
	Asymmetry		FBM 7
	Peak Tailing		FBM 8
Raptor (Corshell) Biphenyl 100*2.1 1.8 µm MeOH	Width 5%	min	RCBM 2
	Retention Factor /BPA		RCBM 5
	Asymmetry		RCBM 7
	Peak Tailing		RCBM 8
	Width 5%	min	CC18M 2

Cortecs C18 + (corshell) 100*2.1 1.6 µm MeOH	Retention Factor /BPA		CC18M 5
	Asymmetry		CC18M 7
	Peak Tailing		CC18M 8

Table S3: Predicted clearance indices for the 15 bisphenols determined using three QSAR models based on either molecular descriptors, a combination of molecular and chromatographic descriptors or chromatographic descriptors. Leverage values were determined with R software package olsrr, leverage higher than threshold given by Olsrr were identified the asterisk. Predicted CI were compared with the observed clearance indices of the 15 bisphenols determined on five perfused human placentae.

		Molecular descriptors		Molecular and chromatographic descriptors		Chromatographic descriptors	
Bisphenol	Observed CI	Predicted CI	Leverage	Predicted CI	Leverage	Predicted CI	Leverage
33BPA	0.65	0.69	0.03	0.62	0.06	0.67	0.05
	0.82		0.03		0.05		0.04
	0.66		0.03		0.06		0.05
	0.73		0.03		0.06		0.04
	0.75		0.03		0.05		0.04
BP44	0.75	0.63	0.04	0.59	0.08	0.63	0.17
	0.78		0.04		0.08		0.16
	0.47		0.07		0.13		0.27*
	0.57		0.05		0.11		0.22*
	0.74		0.04		0.09		0.17
BPA	0.70	0.64	0.02	0.76	0.03	0.62	0.03
	0.85		0.02		0.03		0.03
	0.81		0.02		0.03		0.03
	0.85		0.02		0.03		0.03
	0.85		0.02		0.03		0.03
BPAF	0.42	0.49	0.24*	0.51	0.17	0.57	0.14
	0.49		0.21*		0.15		0.12
	0.60		0.17*		0.12		0.10
	0.54		0.19*		0.13		0.11
	0.57		0.18*		0.13		0.10
BPAP	0.48	0.39	0.01	0.63	0.07	0.60	0.04
	0.53		0.01		0.06		0.04
	0.62		0.01		0.05		0.03
	0.61		0.01		0.06		0.03
	0.61		0.01		0.06		0.03
BPB	0.71	0.64	0.02	0.74	0.04	0.83	0.10
	0.87		0.02		0.03		0.08
	0.88		0.02		0.03		0.08
	0.83		0.02		0.04		0.09

	0.92		0.02		0.03		0.08
	0.20		0.04		0.17		0.12
	0.24		0.04		0.14		0.10
BPBP	0.28	0.14	0.03	0.21	0.12	0.29	0.09
	0.24		0.04		0.14		0.10
	0.32		0.03		0.11		0.08
	0.27		0.05		0.21*		0.16
	0.37		0.04		0.16		0.12
BPC	0.46	0.43	0.03	0.35	0.13	0.33	0.09
	0.42		0.03		0.14		0.10
	0.44		0.03		0.13		0.10
	0.54		0.03		0.06		0.10
	0.91		0.02		0.04		0.06
BPE	0.70	0.63	0.03	0.77	0.05	0.69	0.08
	0.75		0.02		0.04		0.07
	0.53		0.03		0.06		0.11
	0.71		0.03		0.12		0.07
	0.83		0.03		0.10		0.06
BPF	0.70	0.63	0.03	0.61	0.12	0.77	0.07
	0.78		0.03		0.11		0.07
	0.46		0.05		0.18		0.11
	0.04		0.24*		0.29*		0.29*
	0.05		0.20*		0.23*		0.23*
BPFL	0.08	0.08	0.12	0.06	0.14	0.06	0.14
	0.06		0.16*		0.19		0.19*
	0.09		0.11		0.13		0.13
	0.45		0.07		0.10		0.02
	0.19		0.16		0.25*		0.06
BPM	0.53	0.44	0.06	0.33	0.09	0.39	0.02
	0.47		0.06		0.10		0.02
	0.57		0.05		0.08		0.02
	0.49		0.06		0.10		0.01
	0.40		0.07		0.12		0.01
BPP	0.41	0.43	0.07	0.50	0.12	0.32	0.01
	0.45		0.07		0.11		0.01
	0.51		0.06		0.10		0.01
	0.07		0.23*		0.23*		0.23*
	0.07		0.23*		0.22*		0.23*
BPS	0.10	0.08	0.16*	0.08	0.16	0.08	0.16
	0.07		0.23*		0.22*		0.23*
	0.10		0.16*		0.16		0.16
	0.21		0.04		0.13		0.11
BPZ	0.25	0.54	0.04	0.40	0.11	0.32	0.10
	0.44		0.02		0.06		0.05
	0.35		0.03		0.08		0.07

	0.34		0.03		0.08		0.07

<u>Table S4:</u> Molecular descriptors significantly describing the chromatographic descriptors selected in QSAR models based either on chromatographic parameters alone or both chromatographic and molecular. Significant molecular descriptors were identified using ANN model with chromatographic descriptors as output data, molecular descriptors with weight higher than twice the median were considered significant.

	**Significant descriptors included in the model based on chromatographic descriptors**						**Significant chromatographic descriptors included in the model based on chromatographic + molecular descriptors**			
	**T3A 2**	**PFPA 7**	**CC18A 7**	**C8M 7**	**PFPM 7**	**CNM 8**	**C8A 2**	**RCBA 8**	**PFPM 2**	**PFPM 7**
**Molecular weight**										
**Boiling Point**										
**Critical Pressure**	X									
**Critical Temperature**	X							X	X	
**Critical Volume**										
**Gibbs Free Energy**			X							
**Heat Of Formation**	X									
**Henry's Law constant**	X	X	X			X	X	X	X	
**Ideal Gas Thermal Capacity**										
**logP**						X				
**Melting Point**								X		
**Mol Refractivity**										
**Log P Viswanadhan's fragmentation**	X					X				
**Log P Broto's fragmentation**										
**MR Crippen's fragmentation**										
**MR Viswanadhan's fragmentation**										
**Connolly Accessible Area**										
**Connolly Molecular Area**	X				X		X	X	X	X
**Connolly Solvent Excluded Volume**	X	X	X	X	X	X	X	X	X	X
**Number of HBond Acceptors**										
**Mol Refractivity**	X									
**Partition Coefficient**										
**Log P**	X					X				
**Log S**	X									
**pKa1**		X								
**pKa2**			X							
**Balaban Index**										

**Cluster Count**	**X**	**X**		**X**					**X**	
**Molecular Topological Index**										
**Num Rotatable Bonds**										
**Polar Surface Area**	**X**	**X**	**X**	**X**	**X**		**X**	**X**	**X**	**X**
**Radius**							**X**			
**Shape Attribute**										
**Shape Coefficient**									**X**	
**Sum Of Degrees**										
**Sum Of Valence Degrees**						**X**				
**Topological Diameter**			**X**				**X**			
**Total Connectivity**	**X**								**X**	
**Total Valence Connectivity**	**X**								**X**	
**Wiener Index**										
**Heat of formation**	**X**	**X**		**X**	**X**	**X**	**X**	**X**	**X**	**X**
**Total Energy**	**X**	**X**	**X**	**X**	**X**	**X**	**X**	**X**	**X**	**X**
**Electronic Energy**										
**Core-core repulsion**										
**Cosmo Area**	**X**	**X**	**X**	**X**	**X**	**X**	**X**	**X**	**X**	**X**
**Cosmo Volume**										
**Ionisation Potential**	**X**	**X**	**X**	**X**	**X**			**X**		**X**
**Homo Energy**			**X**		**X**					**X**
**Lumo Energy**		**X**	**X**	**X**	**X**	**X**		**X**		**X**
**Dipole moment**	**X**		**X**							
